# Value of Immediate Heart Rate Alteration From Supine to Upright in Differential Diagnosis Between Vasovagal Syncope and Postural Tachycardia Syndrome in Children

**DOI:** 10.3389/fped.2018.00343

**Published:** 2018-11-19

**Authors:** Chunyan Tao, Selena Chen, Hongxia Li, Yuanyuan Wang, Yuli Wang, Ping Liu, Ying Liao, Chunyu Zhang, Chaoshu Tang, Hongfang Jin, Junbao Du

**Affiliations:** ^1^Department of Pediatrics, Peking University First Hospital, Beijing, China; ^2^Division of Biological Sciences, University of California, San Diego, San Diego, CA, United States; ^3^Department of Physiology and Pathophysiology, Peking University Health Sciences Centre, Beijing, China

**Keywords:** vasovagal syncope, postural tachycardia syndrome, orthostatic intolerance, heart rate, acceleration index, 30/15 ratio, children

## Abstract

**Objectives:** To explore the predictive value of immediate heart rate alteration from supine to upright in the differential diagnosis between vasovagal syncope (VVS) and postural tachycardia syndrome (POTS) in children.

**Materials and Methods:** A total of 76 pediatric outpatients or inpatients who visited the Peking University First Hospital from July 2016 to November 2017 were recruited in the study. Among them, 52 patients were diagnosed with VVS and 24 patients were diagnosed with POTS. The differential diagnostic value of acceleration index (AI) and 30/15 ratio was evaluated by the receiver operating characteristic (ROC) curve. An external validation test was performed in another 46 patients.

**Results:** Compared with the cases in the VVS group, patients in the POTS group had a significantly increased AI but a decreased 30/15 ratio (33.495 ± 8.472 vs. 23.440 ± 8.693, *p* < 0.001; 0.962 ± 0.067 vs. 1.025 ± 0.084, *p* = 0.002; respectively). The ROC curves showed that AI and 30/15 ratio were useful for differentiating POTS from VVS. A cut-off value of AI set at 28.180 yielded a sensitivity of 79.2% and a specificity of 73.1%. A cut-off value of 30/15 ratio set at 1.025 yielded a sensitivity of 87.5% and a specificity of 61.5%. A combined use of these two indices improved the sensitivity to 95.8% when either AI or 30/15 was used, and specificity to 80.8% with the use of both AI and 30/15 at the same diagnosis. The external validation test showed that the positive and negative predictive values of the AI and 30/15 ratio were 77.3 and 79.2%, and 72.0 and 81.0%, respectively. The positive predictive value increased to 87.5% when both the AI and 30/15 ratio cut-off values were used together.

**Conclusions:** The AI and 30/15 ratio, which are easy to perform and non-invasive, have proper sensitivity and specificity to differentiate patients with POTS from those with VVS. The combination of these two indices significantly improves the predictive value.

## Introduction

Orthostatic intolerance (OI) is a combination of signs and symptoms that are elicited by standing upright and relieved by recumbency, and it can be divided into acute and chronic subtypes. Acute OI manifests as syncope due to global cerebral hypoperfusion resulting in a sudden and transient unconsciousness, loss of muscular tension and failure to maintain an active position. Chronic OI symptoms include dizziness, headache, palpitations, chest tightness, blurred vision, fatigue, pre-syncope, etc. (1). Vasovagal syncope (VVS) and postural tachycardia syndrome (POTS) are the common causes of OI in children and represent acute and chronic OI, respectively (2, 3). Clinically, manifestations of VVS and POTS have many aspects in common. An obvious increase in heart rate during standing up test or head-up tilt test (HUTT) could be observed among children with VVS (4). Meanwhile, syncope could be the main symptom in children with POTS (5). HUTT, which has been regarded as a valid method to diagnose VVS and POTS (6, 7) is time-consuming and often makes patients feel uncomfortable and even induces cardiac arrest or other critical situations. Thus, its use has been restricted to outpatients and elementary hospitals. Subsequently, much interest has been generated for finding simple, acceptable, safe and efficient alternatives to differentiate VVS and POTS for outpatient physicians and elementary hospital physicians. Zhang et al. found that plasma hydrogen sulfide could be used to identify VVS and POTS, with a sensitivity of 90% and a specificity of 80% when the cut-off value was set at 98 μmol/L (8). Meanwhile, serum iron could also be used to differentiate VVS and POTS, with a sensitivity of 93% and a specificity of 65% when the cut-off value was set at 11.8 μmol/L (9). However, both methods were invasive and failed to achieve real-time results, leading to limitations for their clinical use. Many mechanisms have respectively been described in patients with VVS and POTS. Increased vagal activity and/or decreased sympathetic activity, hypovolemia, vasomotor dysfunction and abnormal Bezold-Jarisch reflex participate in the pathogenesis of VVS (10–14). While, POTS occurs as a result of augmented sympathetic function and hyperadrenergic stimulation, autonomic denervation, hypovolemia, deconditioning, and hypervigilance (14–18). While, alternative autonomic dysfunction seems to be involved in the genesis of both VVS and POTS. Therefore, indicators, which can reflect autonomic function and be detected non-invasively and easily, will be applied more broadly to distinguish POTS from VVS.

The acceleration index (AI) and 30/15 ratio represent immediate heart rate alteration from supine position to standing. Previous evidence favored the concept that the AI reflected the function of the sympathetic system and the 30/15 ratio reflected the function of the vagal system (19, 20). Therefore, we hypothesized that immediate heart rate alteration from supine position to standing would become a non-invasive and convenient real-time way to identify patients with VVS or POTS.

## Materials and methods

**Figure 1:**
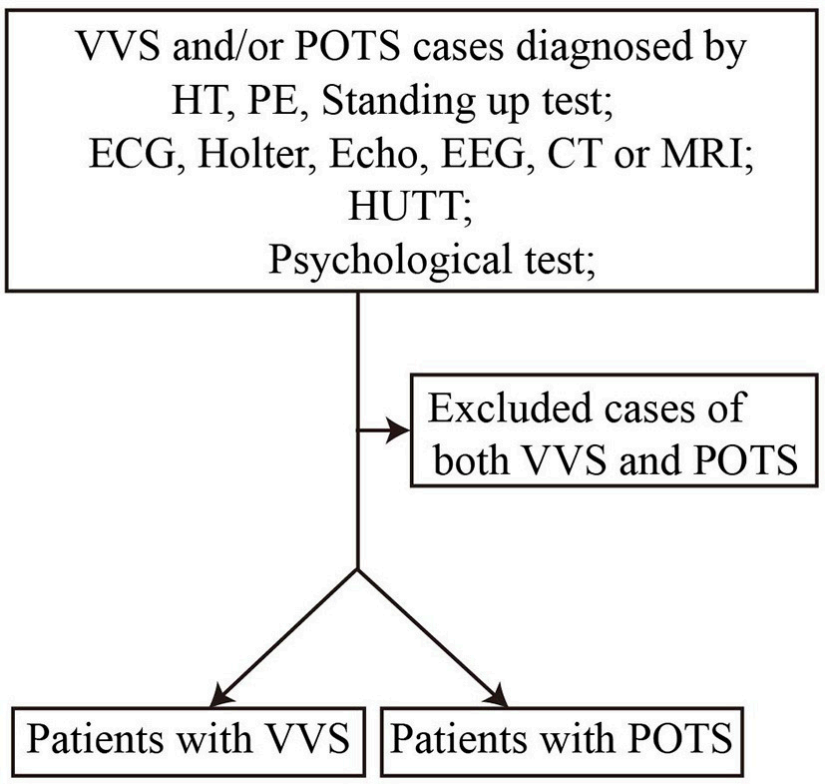
Flow chart of enrollment of study population. VVS, vasovagal syncope; POTS, postural tachycardia syndrome; HT, history taking; PE, physical examination; ECG, electrocardiogram; Echo, echocardiogram; EEG, electroencephalogram; CT, computed tomography; MRI, magnetic resonance imaging; HUTT, head-up tilt test.

We conducted a retrospective study to explore the predictive value of the AI and 30/15 ratio. Between July 2016 and November 2017, 76 patients diagnosed with VVS or POTS in the Department of Pediatrics at Peking University First Hospital were recruited in the study after exclusion of 9 cases suffering both from VVS and POTS [(21–23), Figure 1]. Among them, 52 were diagnosed with VVS, 21 males and 31 females, at an average age of 12.4 ± 1.7 years. Twenty-four patients were diagnosed with POTS, 14 males and 10 females, at an average age of 12.3 ± 2.2 years. Next, a prospective study was performed from December 2017 to May 2018 to validate the predictive values of the AI and 30/15 ratio. Another 46 patients diagnosed with VVS or POTS in the Department of Pediatrics at Peking University First Hospital were recruited in the validation study after exclusion of 4 cases suffering both from VVS and POTS. Among them, 24 patients were diagnosed with VVS and 22 patients were diagnosed with POTS and they were assigned to receive examinations including AI and 30/15 ratio. Twenty-two cases had an increased AI (>28.180), 10 males and 12 females, at an average age of 12.4 ± 2.2 years, while 25 cases had a decreased 30/15 ratio (< 1.025), 8 males and 17 females, at an average age of 11.9 ± 2.2 years. This study was approved by the ethics committee of Peking University First Hospital and written informed consent was obtained from the parents/guardians of participants of this study.

The diagnosis of VVS depends on the following criteria: usually among school-aged children or adolescents; a history of acute episodes of syncope or presyncope; presence of predisposing factors in most of the patients, like prolonged standing and emotional stress; a positive response during the HUTT, namely, significant hypotension (systolic blood pressure ≤ 80 mmHg, diastolic blood pressure ≤ 50 mmHg or the decrease of mean blood pressure ≥25%), bradycardia (heart rate < 75 beats/min in 4–6 years old, < 65 beats/min in 6–8 years old, and < 60 beats/min in children older than 8 years old), sinus arrest, or II° or larger than II° atrioventricular block and asystole longer than 3 s; and the exclusion of other diseases that may likely cause syncope (21, 22).

Criteria of POTS in children mainly consist of the following: usually among school-aged children or adolescents; suffering from chronic OI symptoms; normal supine heart rate; a positive response during the standing up test or HUTT (an increase of heart rate ≥40 beats/min or a maximum heart rate >120 beats/min during the standing up test or HUTT, with a decrease in blood pressure < 20/10 mmHg); and the exclusion of other diseases that may likely cause OI symptoms (21–23).

The symptom scores of POTS were determined by the presence of the following nine typical symptoms, including syncope, dizziness, headache, blurred vision, chest tightness, palpitation, nausea, tremors and sweating. Each symptom was equally and numerically counted based on its frequency (0 score, OI symptoms never occur; 1 score, OI symptoms once per month; 2 scores, OI symptoms between two and four times per month; 3 scores, OI symptoms between two and seven times per week; 4 scores, more than once per day), and the total scores were calculated by summing all of the scores (24).

Protocol for standing up test and HUTT: Drugs capable of influencing autonomic nervous system activity were avoided for at least 3 days before the test. The environment was quiet, dimly lit, and warm. For standing up test, electrocardiograms, heart rate and blood pressure were continuously monitored by a Dash 2000 Multi-Lead Physiological Monitor (General Electric, Schenectady, New York). After 10–20 min of supine rest, patients were asked to stand up and remain in upright position for 10 min, or until the patient could no longer persist in finishing the test. For HUTT, electrocardiograms, heart rate and blood pressure were continuously monitored by Finapres Medical System (FMS, FinometerPRO, FMS Company, Netherlands). Patients were positioned on a tilt table (HUT-821, Beijing Juchi, China) at 60° passively after 10–20 min of supine rest and remained in tilt position for 45 min, or if a positive response was observed the test was terminated ahead. Resuscitation facilities were prepared before the test (21). All patients received both standing up test and HUTT within 3 days.

Immediate heart rate alteration from supine position to standing was recorded and calculated as follows (25, 26):

A (ms) = the mean length of R-R interval during 15 s before changing positions;
B (ms) = the first shortest R-R interval after changing positions;
RR15 (ms) = the length of 15th R-R interval after changing positions;
RR30 (ms) = the length of 30th R-R interval after changing positions;
AI = [(A-B)/A] × 100;
30/15 ratio = RR30/RR15.

SPSS 21.0 statistical software (SPSS Inc, Chicago, Illinois) was used for data analyses, and the data was expressed as mean ± SD. The Shapiro-Wilk test was applied to evaluate the normality of the distribution for continuous data before statistical analyses. Two independent samples *t-*test was used to compare the continuous variables meeting normal distribution between 2 groups. The Mann-Whitney *U*-test was performed if the distribution was skewed. A chi-square test was performed to compare differences in the proportion of categorical variables between 2 groups. The Pearson correlation test was used to examine the correlation between normal distribution indices and the Spearman correlation test was used to examine the correlation between non-normal distribution indices. The receiver operating characteristic (ROC) curve was used to explore the probability of correctly discriminating patients with POTS from those with VVS by using the AI and 30/15 ratio. A *p*-value < 0.05 was statistically significant.

## Results

There were no obvious differences in baseline characteristics between the VVS group and the POTS group (Table 1). Compared with the VVS group, the POTS group had a significantly increased AI but decreased 30/15 ratio (Table 2). The positive response time of VVS patients in the HUTT was 26.1 ± 15.1 min, and at the positive response time in the HUTT, their heart rate, systolic and diastolic blood pressure were respectively 86 ± 31 beats/min, 68 ± 11 mmHg and 41 ± 8 mmHg. For POTS patients, their symptom scores were 6 ± 3 points.

**Table 1:** Baseline characteristics of patients diagnosed with VVS or POTS.

**Items**	**Cases (*n*)**	**Gender (*n*, male/female)**	**Age (years)**	**Weight (kg)**	**Height (cm)**	**Supine SBP (mmHg)**	**Supine DBP (mmHg)**
VVS group	52	21/31	12.4 ± 1.7	47.8 ± 9.4	158 ± 11	111 ± 8^*^	67 ± 6
POTS group	24	14/10	12.3 ± 2.2	48.8 ± 12.3	160 ± 13	110 ± 9	66 ± 8
*χ^2^*/*t*/*Z*-value	–	2.129	0.069	−0.355	−0.589	−0.112	0.802
*p*-value	–	0.145	0.945	0.724	0.558	0.911	0.425

*VVS, vasovagal syncope; POTS, postural tachycardia syndrome; SBP, systolic blood pressure; DBP, diastolic blood pressure*;

* *Continuous variables with non-normal distribution*.

**Table 2:** Immediate heart rate alteration from supine position to standing in VVS and POTS groups.

**Items**	**Cases (*n*)**	**A (ms)**	**B (ms)**	**RR15 (ms)**	**RR30 (ms)**	**AI**	**30/15 ratio**
VVS group	52	779.428 ± 109.068	591.067 ± 67.639	631.019± 89.429^*^	644.087 ± 86.241	23.440 ± 8.693	1.025 ± 0.084
POTS group	24	832.206 ± 159.840	544.201 ± 77.203	595.624 ± 99.026	570.079 ± 85.785	33.495 ± 8.472	0.962 ± 0.067
*t*/*Z*-value	–	−1.683	2.684	−1.185	3.483	−4.724	3.187
*p-*value	–	0.096	0.009	0.236	0.001	< 0.001	0.002

*VVS, vasovagal syncope; POTS, postural tachycardia syndrome; AI, acceleration index*;

* *Continuous variables with non-normal distribution*.

**Figure 2:**
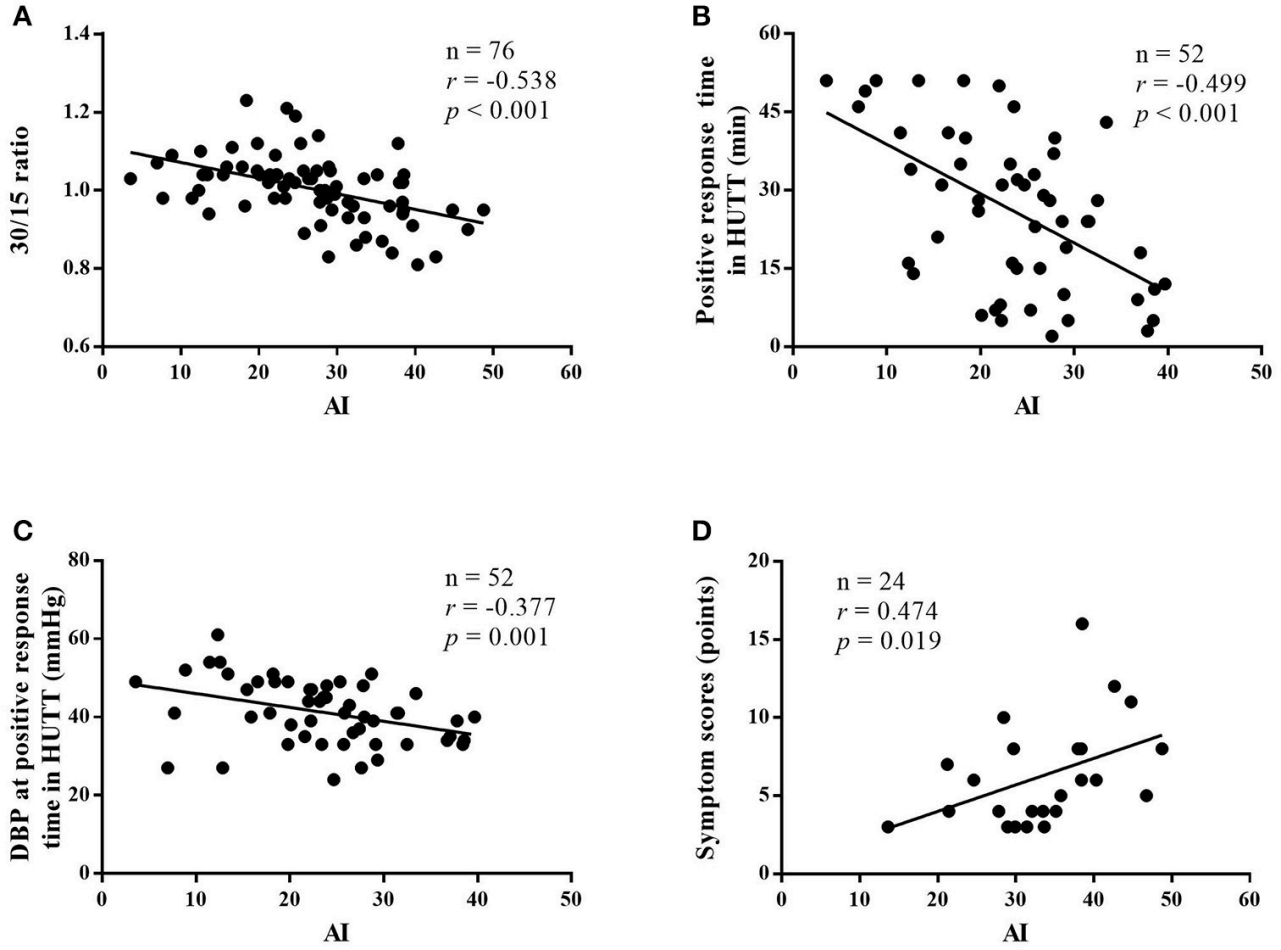
Correlations between immediate heart rate alteration indices from supine position to standing and the severity of diseases. Correlation between the AI and 30/15 ratio of patients with VVS and POTS **(A)**, correlation between the AI and positive response time in HUTT in patients with VVS **(B)**, correlation between the AI and diastolic blood pressure at positive response time in HUTT in patients with VVS **(C)**, and correlation between the AI and symptom scores in patients with POTS **(D)**. VVS, vasovagal syncope; POTS, postural tachycardia syndrome; AI, acceleration index; DBP, diastolic blood pressure; HUTT, head-up tilt test.

Among all of the subjects, AI had a negative correlation with 30/15 ratio (*r* = −0.538, 95% confidence interval (CI), −0.684 to −0.349, *p* < 0.001; Figure 2A). In the VVS group, the AI negatively correlated with the positive response time in the HUTT (*r* = −0.499, 95% CI, −0.684 to −0.254, *p* < 0.001; Figure 2B) and negatively correlated with the diastolic blood pressure at positive response time (*r* = −0.377, 95% CI, −0.589 to −0.116, *p* = 0.006; Figure 2C). In the POTS group, the AI positively correlated with the symptom scores (*r* = 0.474, 95% CI, 0.075 to 0.742, *p* = 0.019; Figure 2D).

**Figure 3:**
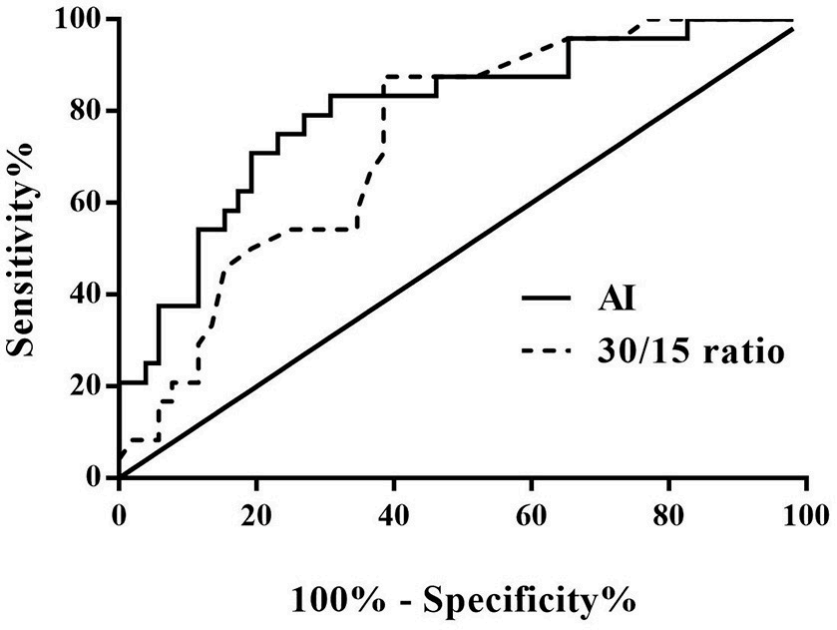
ROC curve of the predictive values of AI and 30/15 ratio for distinguishing patients with POTS from those with VVS. The *y*-axis represents the sensitivity to predict the diagnosis of POTS; the *x*-axis represents the false-positive rate (100% - specificity%). The *black straight line* located to the coordinate 45° of the chart represents the reference line, which indicates that the sensitivity and the false-positive rate are equal. The area under the curve of AI was 0.801 with a 95% CI 0.693 to 0.909 (*p* < 0.001) and the area under the curve of the 30/15 ratio was 0.738 with a 95% CI 0.626 to 0.851 (*p* = 0.001). VVS, vasovagal syncope; POTS, postural tachycardia syndrome; AI, acceleration index.

The ROC curve was used to evaluate the predictive value of the AI and 30/15 ratio in the differential diagnosis between VVS and POTS. When the AI at 28.180 was regarded as the cut-off value, the predictive sensitivity and specificity were 79.2 and 73.1%, respectively. When the 30/15 ratio cut-off value was set at 1.025, the predictive sensitivity and specificity were 87.5 and 61.5%, respectively (Figure 3).

When both the AI and 30/15 ratio cut-off values were used together, the specificity was 80.8% and the sensitivity was 70.8%. When either AI or 30/15 ratio cut-off value was used at the same diagnosis, the sensitivity was highly improved to 95.8%, however, the specificity decreased to 53.8%.

**Figure 4:**
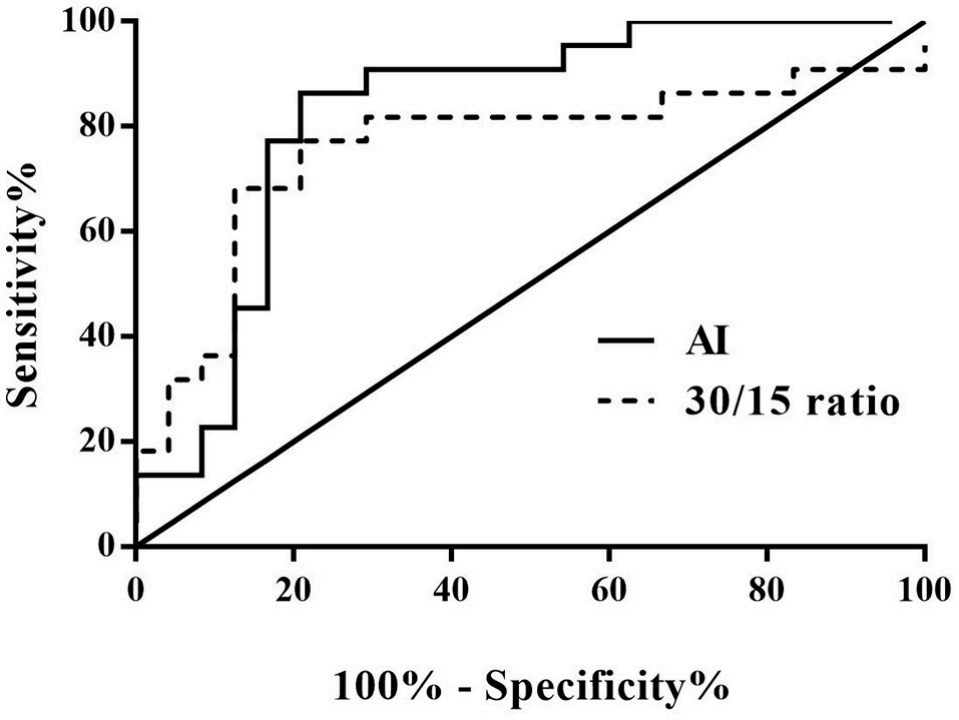
ROC curve of AI and 30/15 ratio in the external validation test. The *y*-axis represents the sensitivity to predict the diagnosis of POTS; the *x*-axis represents the false-positive rate (100% - specificity%). The *black straight line* is located to the coordinate 45° of the chart represents the reference line, which indicates that the sensitivity and the false-positive rate are equal. The area under the curve of AI was 0.826 with a 95% CI 0.699 to 0.953 (*p* < 0.001) and the area under the curve of the 30/15 ratio was 0.760 with a 95% CI 0.607 to 0.912 (*p* = 0.003). VVS, vasovagal syncope; POTS, postural tachycardia syndrome; AI, acceleration index.

From the validation test, no significance was found in baseline characteristics between high AI (>28.180) and low AI (≤ 28.180) cases, nor between decreased 30/15 ratio (< 1.025) and increased 30/15 ratio (≥1.025) subjects (Table 3). Clinically, 22 cases were diagnosed as POTS and 24 cases as VVS. Figure 4 and Table 4 demonstrated the verified efficacy of AI and 30/15 ratio. The positive predictive value increased to 87.5%, the negative predictive value was 73.3% and the diagnostic value was 78.3% when both the AI and 30/15 ratio cut-off values were used together. When either the AI or 30/15 ratio was used at the same diagnosis, the positive and negative predictive values became 62.5 and 85.7%, and the diagnostic value became 69.6%, accordingly.

**Table 3:** Baseline characteristics of the external validation test participants.

**Items**		**Cases (*n*)**	**Gender (*n*, male/female)**	**Age (years)**	**Weight (kg)**	**Height (cm)**
AI	>28.180	22	10/12	12.4 ± 2.2	52.2 ± 12.4	160 ± 12
	≤ 28.180	24	9/15	12.1 ± 1.6	50.6 ± 9.5	163 ± 10
	*χ^2^*/*t*-value	–	0.300	0.419	0.513	−0.727
	*p-*value	–	0.584	0.677	0.611	0.471
30/15 ratio	< 1.025	25	8/17	11.9 ± 2.2	49.9 ±13.0	158 ± 12
	≥1.025	21	11/10	12.7 ± 1.4^*^	53.1 ± 7.8	164 ± 9
	*χ^2^*/*t*/*Z*-value	–	1.955	−1.197	−0.992	−1.972
	*p*-value	–	0.162	0.231	0.327	0.055

*AI, acceleration index*;

* *Continuous variables with non-normal distribution*.

**Table 4:** The predictive values of AI and 30/15 ratio in the external validation test.

**Items**	**Cut-off value**	**Clinical diagnosis**	
		**POTS (*n* = 22)**	**VVS (*n* = 24)**
AI	>28.180 (*n* = 22)	17	5
	≤ 28.180 (*n* = 24)	5	19
30/15 ratio	< 1.025 (*n* = 25)	18	7
	≥1.025 (*n* = 21)	4	17

*AI, acceleration index; VVS, vasovagal syncope; POTS, postural tachycardia syndrome*.

## Discussion

In the present study, we found that the AI was significantly higher but 30/15 ratio was significantly lower in VVS group than that in POTS group, and both of them showed usefulness in the differential diagnosis between POTS and VVS. With 28.180 as the cut-off value of AI, its sensitivity and specificity in the differential diagnosis between POTS and VVS were 79.2 and 73.1%, respectively. Meanwhile, with 1.025 as the cut-off value of 30/15 ratio, its sensitivity and specificity were 87.5 and 61.5%, respectively. Furthermore, combined use of the 2 indices could respectively improve the specificity and sensitivity. More importantly, our external validation test verified the above results.

The rationale that AI and 30/15 ratio could distinguish POTS from VVS has not been fully understood. The AI and 30/15 ratio reflected the immediate heart rate alteration from supine position to standing, which were firstly used to evaluate autonomic nervous function of patients with diabetic neuropathy in the 1970s. Sympathetic activity increased along with the augmentation of AI; whereas, vagal activity increased with the increase in 30/15 ratio (19, 20, 27, 28). Some studies have revealed that sympathetic activation was involved in the pathogenesis of POTS (15–17, 29, 30), nonetheless, sympathetic dysfunction was involved in the pathogenesis of VVS (10–13, 31, 32). The above facts might, in part, help in comprehending our findings in the present study that AI and 30/15 ratio were useful for distinguishing POTS from VVS.

The AI and 30/15 ratio in the differential diagnosis between POTS and VVS have distinct advantages as compared with those found in previous studies. Previously, in the biomarker studies in differential diagnosis between POTS and VVS, plasma hydrogen sulfide (8) and serum iron (9) were elucidated, respectively, and the results showed that they played roles in differentiating POTS from VVS. However, their clinical use was limited not only for the invasive process for obtaining blood samples but for the non-real time results. Comparatively, the present study showed that AI and 30/15 ratio were non-invasive, easy to perform, inexpensive and we could get the real time results in clinics.

In our study, we showed that the AI and 30/15 ratio were negatively correlated, which indicated that the imbalance between sympathetic function and para-sympathetic function might be involved in the mechanisms for VVS and POTS. Meanwhile, the results of the present study showed that AI correlated with the severity of the cases. For instance, the results showed that in the VVS group, during the positive response of the tilt test, AI was negatively correlated with both the positive response time and the diastolic blood pressure. The Bezold-Jarisch reflex is a crucial part in the pathogenesis of VVS (33, 34) in which sympathetic nervous activity plays an important role. Increased sympathetic nervous activity leading to vigorous contraction of the hypovolemic ventricle during the HUTT activates the vagal afferents and the activation of vagal nervous system triggers an inhibitory response, resulting in hypotension and/or bradycardia. We may speculate that the stronger sympathetic nervous activity is, the stronger reflective inhibitory response becomes, and consequently positive response occurs earlier and blood pressure gets lower. In the POTS group, AI was positively correlated with symptom scores, which once again illuminated that the higher sympathetic nervous activity was, the more severe symptoms of POTS children were (35).

This study was conducted among children at the mean age of 12.0 years, nevertheless, the fact is the function of autonomic nervous system varies with the changes of age and it is also impacted by many other factors, such as endocrine hormones, weight and physical activities (36, 37). As a result, characteristics of the present sample might limit generalizability to other populations. In the future we need to conduct large sample-sized multicenter studies to validate the clinical value of AI and 30/15 ratio in clinics.

In conclusion, the AI and 30/15 ratio differed significantly between VVS and POTS patients. Moreover, the AI was correlated with the severity of these two diseases. The AI and 30/15 ratio could help in the differential diagnosis between VVS and POTS easily and quickly. However, further studies among broader populations are needed to extrapolate the results.

## Author contributions

CyT had primary responsibility for the protocol development, patient enrollment, data collecting, preliminary data analysis and writing the manuscript. SC assisted with data analysis and critical revision for important content, wrote the draft and edited the draft. HL and YYW assisted with analyzing the data and revising important content. YLW, PL, YL, and CZ took the responsibility to complete the head-up test or head-up tilt test. CsT and HJ gave important advice on the subject. JD supervised the design and execution of the study, checked the data analysis, contributed to the writing of the manuscript and had a final approval of the manuscript submitted. All authors have read and approved the final manuscript and assumed full responsibility for its contents.

### Conflict of interest statement

The authors declare that the research was conducted in the absence of any commercial or financial relationships that could be construed as a potential conflict of interest.

## Abbreviations

OI: Orthostatic intolerance
VVS: Vasovagal syncope
POTS: Postural tachycardia syndrome
HUTT: Head-up tilt test
AI: Acceleration index
ROC: Receiver operating characteristic
CI: Confidence interval.
